# Understanding undergraduate students’ eHealth usage and views of the patient-provider relationship

**DOI:** 10.1371/journal.pone.0266802

**Published:** 2022-04-14

**Authors:** Michelle Anne Reyes, Heather D. Vance-Chalcraft

**Affiliations:** Department of Biology, East Carolina University, Greenville, North Carolina, United States of America; Imam Abdulrahman Bin Faisal University, SAUDI ARABIA

## Abstract

eHealth has grown exponentially alongside technology and has become widely accessed by some populations, but little is documented about how undergraduate students use eHealth or perceive their eHealth literacy. As access to online information and non-traditional options for interacting with providers has increased, patient views of the provider-patient relationship may also be changing. This study evaluates how frequently undergraduates use eHealth, how they perceive their ability to use eHealth appropriately, and how they view their patient-provider relationships. A mixed methods approach was used to address the research questions, with quantitative data from a survey and qualitative data from follow-up interviews of twelve of the survey respondents. The survey was distributed to over 650 undergraduate students in introductory biology laboratory courses for students of all fields of interest at one university. Based on 527 survey responses and 12 interviews, students reported commonly using eHealth but being skeptical of telehealth appointments. Although students generally felt capable of finding and interpreting eHealth sources, they were not strongly confident in their ability to do so. Use of eHealth was not seen as altering the patient-provider relationship, but students expressed a desire for their physician to act more as a counselor or advisor than a guardian. Students from minority populations were more likely to use eHealth in comparison to their peers. In addition, student comfort with their provider differed by race and ethnicity, as well as whether they shared the same gender identity as their provider. This research highlights how undergraduate students, who are often making medical decisions for themselves for the first time as adults, access health information and view the patient-provider relationship differently than the traditional guardian or paternalistic model. In addition, having diverse, culturally competent medical providers are critical for students to develop the relationship with their provider that they desire.

## Introduction

eHealth includes health services and information made available through the internet and related technologies [1]. Within the past few decades, eHealth has fundamentally changed health communication between medical professionals and patients by offering an avenue of communication outside of the medical office [2]. In addition, eHealth allows individuals to access and research health information [3]. Previous studies have shown that women and young adults are more likely to use eHealth compared to other populations, while some patients may avoid using eHealth based on their time availability, doubt of information accuracy, or inability to search health related information on their own. Thus, there is a “digital divide” between online and offline health information seekers [2,4–8].

While the ability to access eHealth provides an opportunity for some individuals to have greater knowledge about their medical decisions, this benefit cannot be realized if information is not accurately understood and interpreted by the patient. eHealth literacy describes one’s ability to find and use appropriate online health information [9]. Previous studies have shown that the health sources most individuals use are unreliable or inaccurate [10–12] even though expert medical and educational organizations have created credible eHealth sites and offer resources to increase eHealth literacy [13,14].

The current COVID-19 pandemic illustrates the importance of understanding eHealth usage and eHealth literacy as health information has been in high demand, and misinformation has been widely circulated [15]. Moreover, social distancing and other restrictions have limited the options for in person interactions, making video and phone appointments with providers much more commonplace than before [16,17]. Telehealth offers an avenue of communication between the patient and provider from the convenience and safety of their home and serves as another eHealth resource for individuals.

As access to online information and non-traditional options for interacting with providers has increased, patient views of the provider-patient relationship may also be changing. Previously four models of the provider-patient relationship were described: a guardian or paternalistic model in which the provider decides actions for the patient; a counselor or advisor (interpretive) model in which the patient is viewed as having some understanding relevant to the medical care; a friend or teacher (deliberative) model; and a technical expert (informative) model in which the patient has control over their medical care while the provider serves as the technical expert [18]. Some of these models, however, may not reflect the relationship well in an era of information gathering by patients, in which the patients may seek a greater sense of ownership over their health decisions [19].

Understanding patients’ comfort levels with their provider may help in determining the perception they have of their medical provider, as well as the type of patient-provider relationship they want. A patient’s level of comfort and trust with their provider is dependent on many factors such as patient needs and whether those needs are met, environmental factors, the patient’s perception of their health issues, demographic differences with their medical provider, and whether they have a prior relationship with the medical provider they are seeing [20,21]. Patients may feel less comfortable with medical providers who have less experience, especially when being treated for a serious disease or condition [21–23].

Little is known about how undergraduate students view the patient-provider relationship in this era of eHealth. These students may be motivated to empower themselves by taking accountability for their own health as many are newly entering adulthood [24]. Undergraduate students are likely to be frequent users of eHealth due to their comfort with convenient, online information [25]. Thus, they may not rely on a health provider for all their health knowledge and may view the patient-provider relationship differently from individuals who are less likely to access eHealth. However, studies have not examined this relationship between eHealth use and views of the patient-provider relationship among undergraduate students. It has been hypothesized that individuals majoring in health fields would have greater perceived eHealth literacy scores, but that has been contradicted by multiple studies which showed the perceived eHealth literacy of students were similar regardless of their major [10,11,26,27]. Additional research is required to clarify these conflicting findings.

This research asks whether undergraduate students use eHealth, how confident they are in their eHealth literacy, and how they view the patient-provider relationship. Specifically, five questions were evaluated. 1) How much do undergraduate students use eHealth, and does their likelihood of using eHealth vary with specific student characteristics? 2) What is the perceived eHealth literacy of undergraduates, and does it vary with specific student characteristics? 3) What model of the patient-provider relationship do students feel they have, and which model do they want? 4) Do students feel their relationship with their provider has been impacted by their use of eHealth? 5) Does student comfort with their provider, and barriers they perceive to sharing health information with their provider, vary with specific student characteristics?

## Methods

This study used a mixed methods research approach, combining both quantitative data in the form of survey responses and qualitative data in the form of interviews. The goal of the survey was to capture undergraduate students’ demographics, their eHealth usage, perceptions of their eHealth literacy, and information regarding the relationship and level of comfort they have with their provider. Interviews were conducted for the purpose of gaining a better understanding of more specific topics such as eHealth definition and use, barriers which may impede the patient-provider relationship, and the rationale behind the patient-provider relationship the students had or wanted. ECU’s Institutional Review Board (UMCIRB 20–001788) approved this human subjects research. Students were asked to consent to participate in the research study at the beginning of the Qualtrics survey; for those participating in interviews, the consent was requested again verbally and noted by the interviewer, as approved by the IRB. Only students over 18 years of age were eligible to participate.

The study population was undergraduate students over the age of 18 in three laboratory courses at East Carolina University (ECU), Greenville NC. These three courses included General Biology Lab for non-science majors (BIOL 1051), Principles of Biology Lab 1 for science majors (BIOL 1101), and Principles of Biology Lab 2 for science majors (BIOL 1201). These classes had a combined enrollment of 666 students at the time of data collection. As all students (from all fields of study) are required to take one science laboratory course, undergraduates enrolled in these courses were expected to be representative of ECU undergraduates with diverse class ranks, ages, and intended majors. Throughout the manuscript, “patient” refers to undergraduate students as they are our focal population.

### Surveys

A survey (S1 File) was created using a combination of original questions and questions from a validated survey of perceptions of eHealth literacy, called the eHEALS [9]. The original survey questions were formulated to capture respondent demographics (gender identity, race/ethnicity, pre-health intended major, etc.) and information focused on their relationship with their medical provider. Additional questions asked about the information sources individuals used when making decisions about their health and barriers impacting their comfort with their medical providers. Questions were originally written to solicit information that the relevant literature on the subject indicated was most likely to be important, and then the questions were refined and validated iteratively. Five experts reviewed and provided feedback on the wording and importance of the original questions. Thus, they assessed the content validity of the items. After making revisions based on expert feedback, five undergraduates assessed the face validity of the items by completing the original survey questions and then describing how they interpreted each of the questions. Additional revisions were completed for clarification prior to distribution for data collection.

In addition to the author-generated questions, the survey included items related to students’ perceptions of their eHealth literacy. These items were drawn from eHEALS, an 8-item published instrument [9] designed to measure an individual’s perception of their knowledge and ability to find electronic health information and apply that information to health issues [10]. This scale was developed by Norman and Skinner [9] to assess eHealth literacy by providing a general estimate of one’s perceived eHealth-related skills. The items on eHEALS are answered on a 5-point Likert scale ranging from “strongly disagree” to “strongly agree”. eHEALS was incorporated into this research study because it has been widely used and allows for comparison of the results from this study population with those of prior studies. In addition, it was shown to be reliable through item analysis on the 8-item scale at baseline, producing a scale with α = 0.88 [9]. This scale has been validated by two additional research studies which have shown that the internal consistency of the scale was high (α = 0.93 and α = 0.92) and that there were no concerns for multicollinearity (α = 0.94) [28,29]. The eHEALS has been shown to be an appropriate measure for populations of various ages. The author-generated items and eHEALS items were combined into a single survey that was administered electronically through Qualtrics (Qualtrics International) to students in these three lab classes during the first semester block of Fall 2020. Students were offered extra credit for opening the survey, even if they did not consent to participate in the research study, to ensure that students did not feel coerced to participate or bias the sample.

### Survey data analysis

Survey responses were exported from Qualtrics to an Excel (Microsoft Corporation) spreadsheet and filtered to discard incomplete or invalid responses. Surveys which were not completely filled out were discarded and if a student had multiple responses, only the initial survey was retained. Any information that could be used to identify a respondent was removed and each respondent was assigned a unique random number as an identifier. Only deidentified data was used for analyses. The cleaned, deidentified data set was imported into SPSS (IBM) for analysis. All Likert scale results were reversed to make the direction of the scores match the intuitive interpretations (e.g., higher Likert score means higher eHealth usage rather than lower). Two-tailed tests were used for all tests and the threshold for significance for all tests was α less than or equal to 0.05.

The eHealth usage and perceived eHealth literacy of the students was evaluated. To address the first research question about eHealth usage, the average eHealth usage by undergraduates was calculated. Separate ANOVAs were run to determine if the level of student eHealth usage varied with different student characteristics, including having a primary care provider, gender identity, major, race and ethnicity, whether they had taken an introductory health class, and confidence of sharing health information with their medical provider. To address the second research question about perceived eHealth literacy, the mean perceived eHealth literacy was calculated for each student, measured as the mean of their eight eHEALS items. ANOVAs were used to determine whether the perceived eHealth literacy differed based on student major (pre-health or not), whether they had taken an introductory health class, whether they used health websites as a source of health information, or by student race and ethnicity.

In addition, students’ views of the patient-provider relationship, their comfort with their provider, and the barriers they perceived to sharing health information with their provider was evaluated. To address the third research question about patient-provider relationships models, a one-sample t-test was used to determine whether there were significant differences in student preferences for the various patient-provider relationship models, and another t-test to test whether they felt there were differences in how common each patient-physician relationship model was. One-way ANOVAs tested whether the students’ perception of which relationship model was most common or which model they desired. The fourth research question regarding if students perceived their relationship with their provider has been impacted by their use of eHealth sources was addressed through an ANOVA, and was followed by a separate ANOVA to determine if students’ perceptions of the impact of eHealth use on their relationship with their provider differed based on their level of eHealth use. Finally, the fifth research question was evaluated using separate ANOVAs to determine if there were significant differences in the level of comfort a student had when meeting with their physician based on various student characteristics, including having a pre-existing health condition, having a primary care provider, race and ethnicity, and confidence about sharing health information with their medical provider, as well as whether the gender of the medical provider matched that of the undergraduate student. Moreover, ANOVAs were used to determine whether students perceived barriers to sharing health information with their providers based on student race and ethnicity or whether they shared the same gender identity as their provider.

### Interviews

A subset of survey respondents was contacted with an invitation to complete a follow up interview. Respondents were emailed in alphabetical groups based on last names until all interview slots were filled. The goal of the interviews was to provide additional context for the survey results, such as additional factors that can influence the patient-provider relationship and how individuals view eHealth through their own words and experiences. Students were able to describe their patient-provider relationship more fully and the relationship style they wanted to have. The semi-structured interviews were guided by six original discussion questions and followed up with additional clarifying or probing questions, as needed (S2 File). Questions were written to elicit details that may not have been clear from survey responses and could provide relevant context. Three experts provided feedback on the questions. Interviews were conducted through Cisco WebEx in January and February 2021, taking approximately 15–20 minutes each to complete. The first author (a graduate student at the time), who had no affiliation with the course through which the participants were recruited, completed the interviews. Notes were taken during each interview and reviewed afterwards to ensure information captured was appropriate. Students who completed the survey were awarded a $20 Amazon gift card for participation.

Interview notes were analyzed using NVivo (QSSR International). A list of initial codes was generated using information from the published literature and the survey information. Additional codes were added, as needed, based on reviewing the interview notes. After a codebook was created, the author and a colleague coded 25% of transcripts, using the revised codebook. Interrater reliability between these two coders was calculated using kappa coefficient. Disagreements in coding were resolved through discussion and assistance of a third party before revising the codebook, coding another interview, and repeating the kappa coefficient. Once a high inter-rater reliability was achieved (kappa = 0.8653), the remaining interview notes were coded (using the revised codebook provided as S1 Table) by the author and synthesized to identify overarching themes.

## Results

### Surveys

After removing problematic surveys, 527 responses remained for analysis., for a response rate of 79.1%. The survey participants were largely 18–20 years old (89.6%), freshman (56.5%), female (62.4%), and white (61.7%; Table 1). The demographics of the survey respondents were representative of the total undergraduate population at ECU during the Fall 2020 semester (ECU Fact Book), in which approximately 58.3% of students identified as female and 64.1% of students identified as white. Approximately half of the survey respondents intended to major in a pre-health discipline (58.3%), and most did not have a pre-existing health condition (81.2%). 69.1% of respondents shared the same gender identity as their primary care provider.

**Table 1:** Demographic characteristics of survey participants (N = 527).

	Number	%
**Gender Identity**		
Female	329	62.4
Male	196	37.2
Other	1	0.2
Do not wish to answer	1	0.2
**Age**		
18–20	472	89.6
21–24	45	8.5
25+	10	1.9
**Class Rank**		
Freshman	298	56.5
Sophomore	146	27.7
Junior	55	10.4
Senior	28	5.3
**Racial/Ethnic Identity**		
American Indian or Alaska Native	7	1.3
Asian	23	4.4
Black or African American	105	19.9
Hispanic or Latino	39	7.4
Native Hawaiian or Other Pacific Islander	2	0.4
White	325	61.7
Other	21	4
Do not wish to answer	5	0.9
**Taken an Introductory Health Course?**		
Yes	210	39.8
No	219	41.6
Currently Enrolled	98	18.6
**Pre-Existing Health Condition?**		
Yes	88	16.7
No	428	81.2
Do not wish to answer	10	1.9

**Fig 1:**
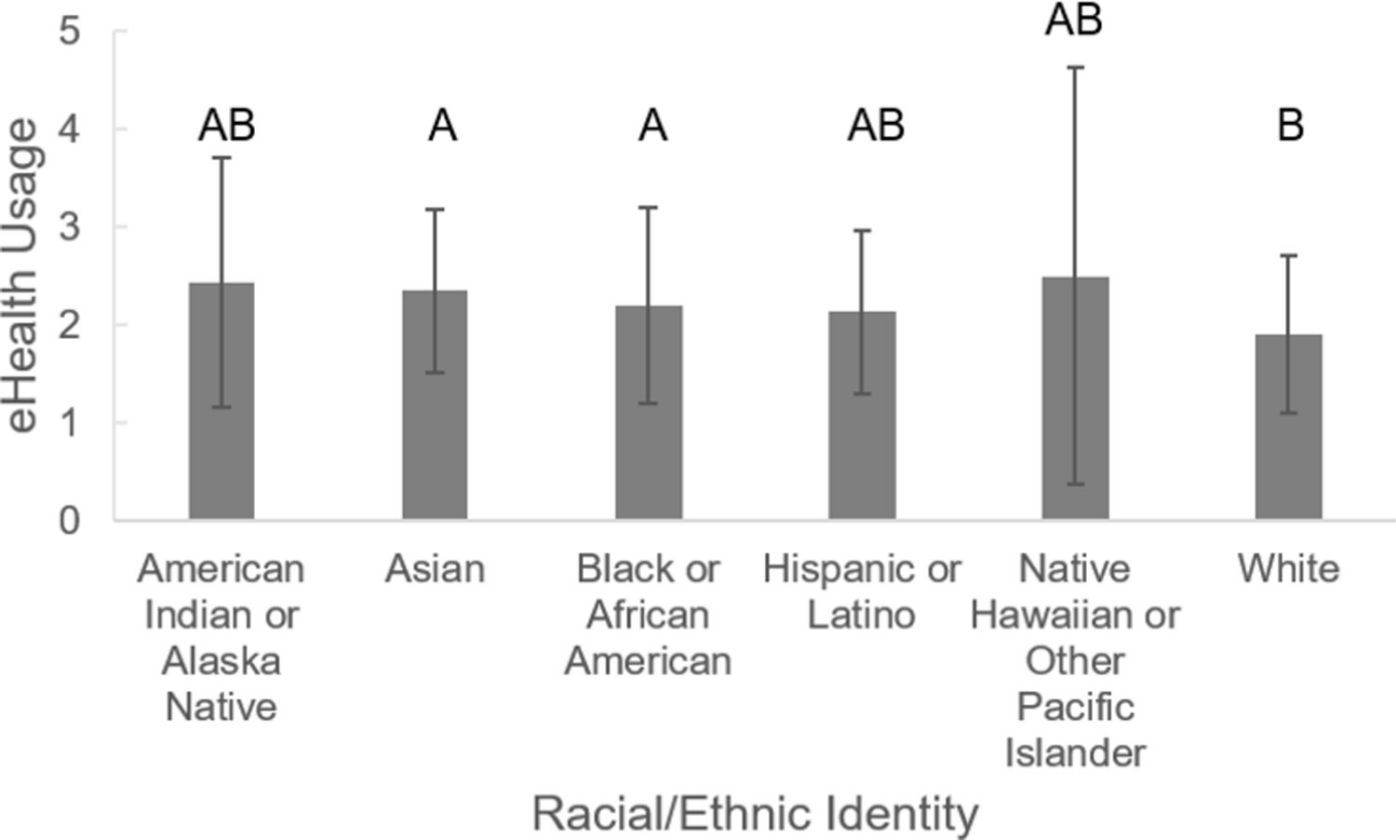
eHealth usage based on racial and ethnic identity. Higher values correspond to higher eHealth usage. Error bars represent standard deviations. Bars that have the same letter(s) above them do not significantly differ from each other.

The first research question evaluated how much undergraduate students use eHealth and how their likelihood of using eHealth varied with specific student characteristics. When asked to rank the information sources they preferred to use when making medical decisions, respondents prioritized information from their medical provider, followed by a family member (Tables 2 and S2). Online sources, such as health information websites, were ranked third and most respondents (74%) indicated they looked up online sources at least occasionally when making decisions about their health (Table 3). Undergraduate students’ eHealth usage did not significantly differ based on whether the student had a primary care provider (F_2, 524_ = 0.868, p = 0.420) or by the gender of the undergraduate student (F_3, 523_ = 0.414, p = 0.743). Levels of eHealth usage also did not differ between respondents in a pre-health intended major and those in other majors (F_1, 525_ = 0.783, p = 0.377). On the contrary, the level of eHealth usage significantly differed based on the race and ethnicity of the undergraduate students (Fig 1; F_7, 519_ = 2.251, p = 0.029). A Fisher’s LSD test of multiple comparisons showed that white students had significantly lower eHealth usage than Asian (p = 0.021) or African American students (p = 0.003). In addition, there was a trend for students who have had an introductory health class to use eHealth more frequently than those who have not had an introductory health class in college or university (F_2, 524_ = 2.786, p = 0.063). Undergraduate students who reported feeling confident sharing information they find online with their providers were more likely to be higher eHealth users (F_1, 525_ = 16.125, p < 0.001).

**Table 2:** Mean ranking by survey respondents for sources they prioritize when making health decisions (1 = top priority, 8 = lowest priority).

Overall Ranking	Mean Rank
1- Medical Provider	1.57
2- Family	2.32
3- Health Information Websites	3.69
4- Friends	4.09
5- Partner	4.27
6- Social Media	5.66
7- Television	6.69
8- Other	7.72

**Table 3:** Number and percent of respondents indicating how often they use online health sources when making decisions about their health.

	Number	%
**All the time**	38	7.2
**Almost every time**	83	15.7
**Occasionally**	269	51
**Very seldom**	122	23.1
**Not at all**	15	2.8

The second research question asked about the perceived eHealth literacy of undergraduates. The eHEALS scores (a measure of perceived eHealth literacy) of respondents who were intending to major in a pre-health discipline did not differ from those who were not pre-health (F_1, 525_ = 1.078, p = 0.300), with an average eHEALS score of 2.71 (S3 Table). eHEALS scores also did not differ significantly based on whether respondents had taken an introductory health class in college/university (F_2, 524_ = 0.416, p = 0.660), whether they used health websites as a source of health information (F_6, 520_ = 0.778, p = 0.587), or by race/ethnicity (F_7, 519_ = 0.500, p = 0.835).

Student views of patient-provider relationship models was evaluated for the third research question. Regarding their medical provider, 83.7% of survey respondents indicated they had a primary care physician. The “counselor/advisor” patient-provider model was reported significantly more often than other models (t_526_ = 67.350, p < 0.001) to reflect the relationship respondents felt was most common with providers, and it was the patient-provider relationship model they wanted most (t_526_ = 79.722, p < 0.001) compared to the other possible models (Table 4). Student levels of eHealth usage impacted their views of which patient-provider relationship model was most common (F_2,524_ = 3.33, p = 0.037), but not their desired relationship model (F_2,524_ = 1.64, p = 0.195). Students who used eHealth often (“all” or “almost all of the time”) were significantly less likely to report that the “Technical Expert” model was most common compared to students who reported using eHealth rarely (“very seldom” or “not at all”).

**Table 4:** Number and percent of respondents indicating how common they think each type of patient-provider relationship is, and which model they want.

	Believe is Most Common		Want	
Relationship Models	Number	%	Number	%
Guardian/Paternalistic	84	15.9	41	7.8
Counselor/Advisor	298	56.5	307	58.3
Technical Expert	122	23.1	120	22.8
Friend/Teacher	23	4.4	59	11.2

To answer the fourth research question about the impact of eHealth on the patient-provider relationship, a narrow majority of students (50.7%) reported that they shared information they find online with their provider and most (74.6%) felt confident doing so. Additionally, respondents indicated that they did not think that accessing online health sources had changed their relationship with their medical provider (67.2%). Students who reported using eHealth rarely (“very seldom” or “not at all”) were more likely to report that accessing online health sources had not changed their relationship with their medical provider compared to students who reported using eHealth often (“all” or “almost all of the time”) (F2, 524 = 14.20, p < 0.0001). Approximately half of frequent eHealth users reported that their relationship with their medical provider had changed.

**Fig 2:**
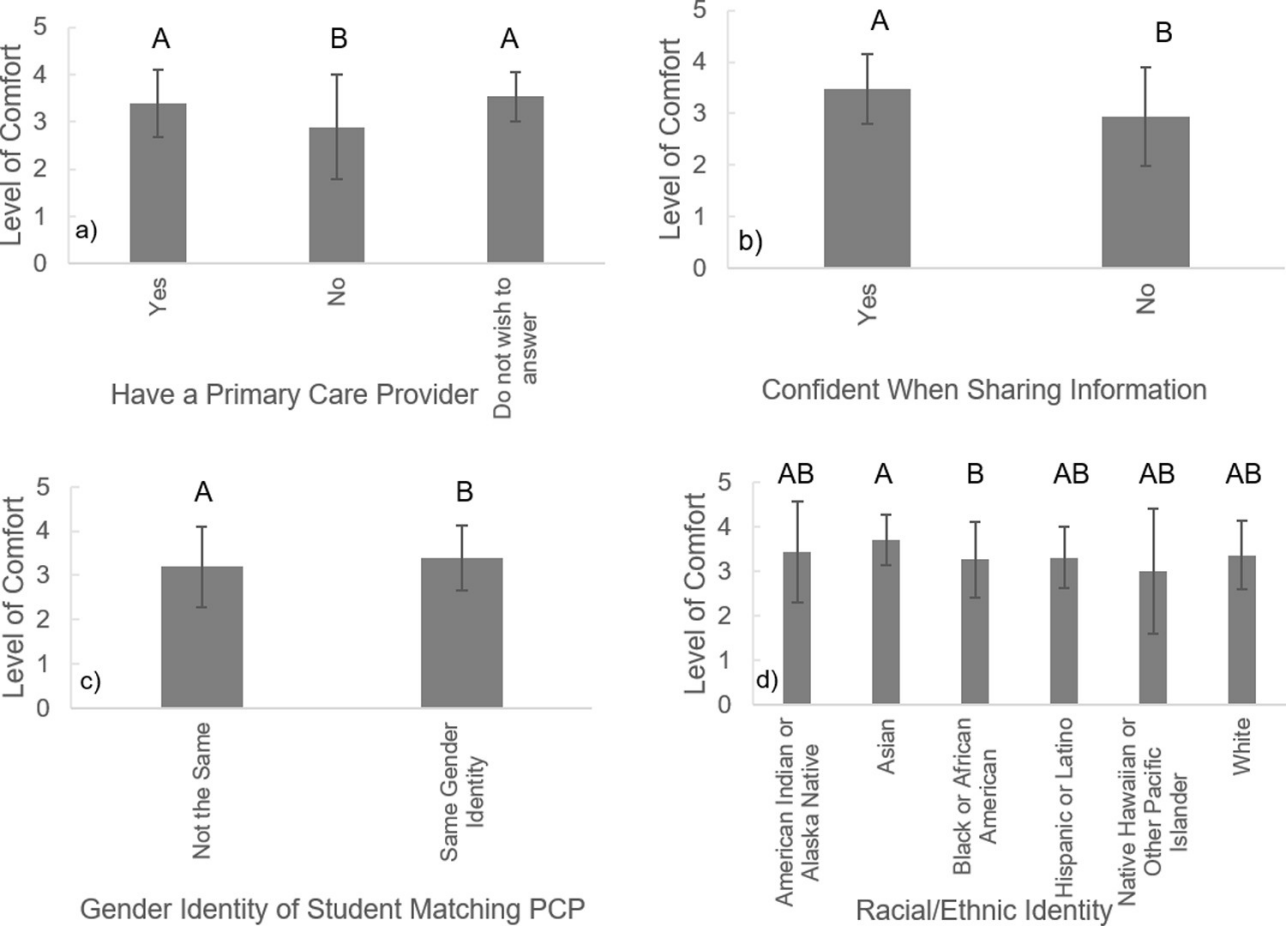
Relationship between the level of comfort (higher values mean a patient is more comfortable) students experience when visiting their medical provider based on a) if they have a primary care provider (PCP), b) if a student feels confident sharing online information they find with their medical provider, c) if the gender of the student and the medical provider match, and d) the racial/ethnic identity of the student. Error bars represent standard deviations. Bars that have the same letter(s) above them within a panel do not significantly differ from each other.

The final research question examined how comfortable students were with their provider, and barriers they perceived to sharing health information with their provider. Most respondents said that they were very comfortable all the time (48.8%) or almost every time (39.7%) they met with their physician. Students who had a pre-existing health condition were not significantly more or less comfortable when meeting with their medical provider (F_2, 523_ = 0.927, p = 0.397) than people without a pre-existing condition. Respondents who had a primary care provider, though, were significantly more comfortable with their provider than those who did not (Fig 2; F_2, 524_ = 13.903, p < 0.001). The level of comfort a student had with their provider varied with the student’s race and ethnicity (Fig 2; F_7, 519_ = 1.973, p = 0.057). A Fisher’s LSD test of multiple comparisons showed that black/African American students were significantly less comfortable with their providers than Asian students (p = 0.016). Students with the same gender identity as their provider felt significantly more comfortable with their provider than those who had a different gender identity (Fig 2; F_1, 525_ = 7.601, p = 0.006). Not surprisingly, respondents who felt confident sharing information from online sources with their physician were also those who felt significantly more comfortable meeting with their provider (Fig 2; F_1, 525_ = 51.468, p < 0.001).

**Fig 3:**
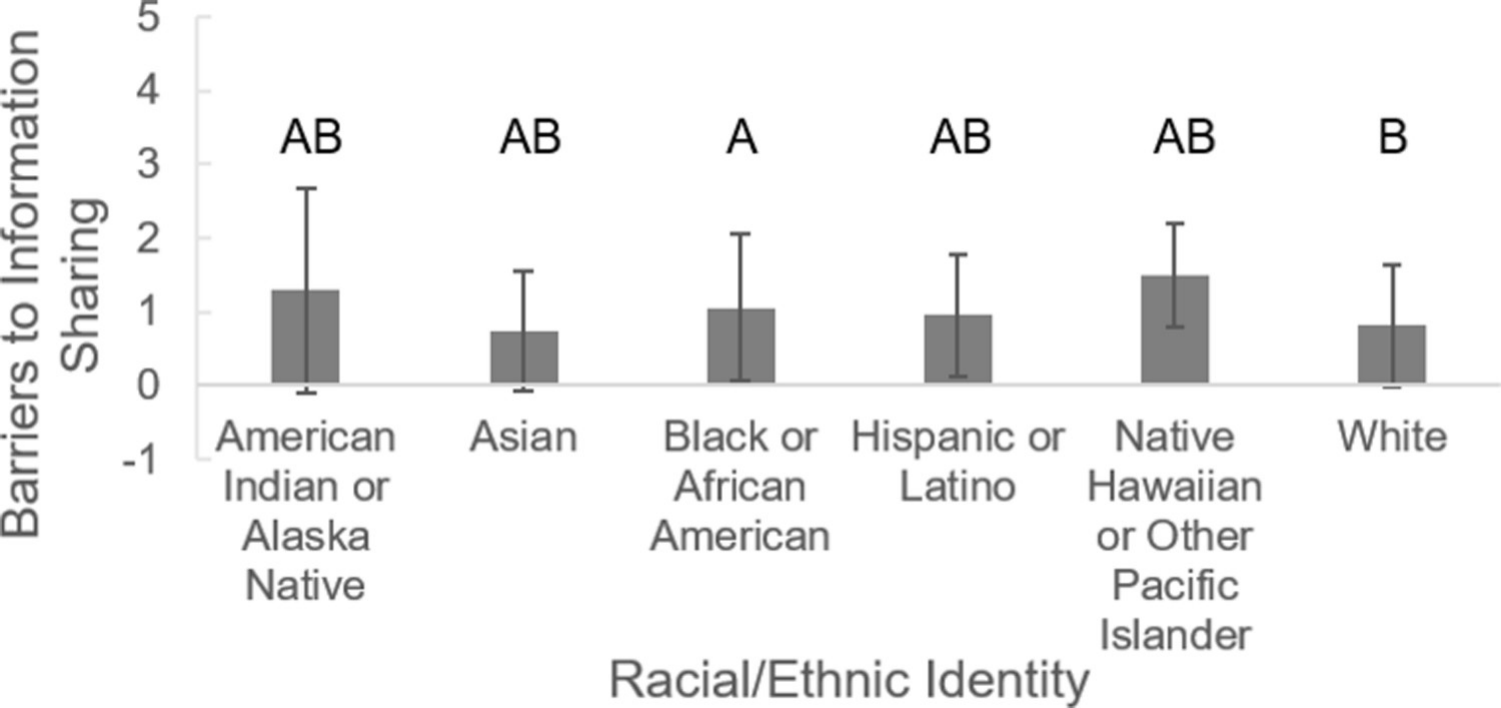
Students of different racial/ethnic identities perceive different frequencies of barriers to sharing health information with their medical provider. Higher values indicate more frequent barriers. Error bars represent standard deviations. Bars that have the same letter(s) above them do not significantly differ from each other.

Whether students reported barriers to sharing health information with their providers varied by student race and ethnicity (Fig 3; F_7, 519_ = 2.630, p = 0.011). A Fisher’s LSD test of multiple comparisons indicated that black/African American students reported more barriers than did white students. Students who shared a gender identity with their physician were equally likely to have reported barriers sharing health information with their provider as students who had a different gender identity from their provider (F_1, 525_ = 1.649, p = 0.200).

### Interviews

The 12 interviewees generally reflected similar demographics as survey respondents (Table 5). The final codebook had nine different codes (S1 Table) which captured aspects of the patient-provider relationship, eHealth sources used, and the influence of the COVID-19 pandemic on eHealth usage and the patient-provider relationship. These nine codes were synthesized into three broad themes: Professionalism, Information Accuracy, and Relationship Status.

**Table 5:** Demographic characteristics of individuals participating in an interview (N = 12).

	Number	%
**Gender Identity**		
Female	9	75
Male	3	25
Other	0	0
Do not wish to answer	0	0
**Age**		
18–20	11	91.7
21–24	1	8.3
25+	0	0
**Class Rank**		
Freshman	10	83.3
Sophomore	1	8.3
Junior	0	0
Senior	1	8.3
**Racial/Ethnic Identity**		
American Indian or Alaska Native	0	0
Asian	1	8.3
Black or African American	2	16.7
Hispanic or Latino	1	8.3
Native Hawaiian or Other Pacific Islander	0	0
White	6	50
Other	2	16.7
Do not wish to answer	0	0
**Pre-Health Intended**		
Yes	9	75
No	3	25
**Taken an Introductory Health Course?**		
Yes	2	16.7
No	7	58.3
Currently Enrolled	3	25
**Pre-Existing Health Condition?**		
Yes	1	8.3
No	10	83.3
Do not wish to answer	1	8.3

Professionalism identifies how undergraduate students valued the professionalism physicians can provide when making decisions about their health, but due to COVID-19 and pandemic restrictions, students indicated how difficult it was to talk and connect to their physician. Students felt that making health decisions was easier with a physician because they are the ‘professionals’ and have better expertise knowledge on health information. Many interviewees, however, recounted telehealth as an inadequate form of communication with their provider especially when they are trying to be diagnosed. An interviewee stated that ‘going in person’ is a better alternative to telehealth because is it more personal and ‘more can be accomplished’ regarding being diagnosed or getting a check-up, although they recognized that telehealth is a safer alternative due to the current pandemic.

A second theme, Information Accuracy, identifies the perception from interviewees that the information they obtained through online sources lacked credibility in comparison to health information they obtained from their medical provider. Several interviewees mentioned that the ability to look up information at home was beneficial for them because it let them learn more about the health topic of concern, but they felt anxious that they may have unwittingly obtained misinformation. Due to this information doubt in online health resources, students tried to verify the information they saw online with their medical provider.

A final theme identified from the interviews was Relationship Length. The length of the relationship between the undergraduate student and their medical provider influenced how much positive encouragement they received from their provider to utilize eHealth as well as how comfortable a student was when meeting with their physician. Interviewees who reported having a long-term relationship with their medical provider (i.e., since childhood) stated that they were more likely to discuss eHealth information with them.

## Discussion

Most students in this study reported accessing online health resources, complementing previous studies that have shown undergraduate students more frequently use eHealth information and resources compared to other age groups [5,7]. Students may become more familiar with online health sources through introductory health courses, as students were more likely to use eHealth if they had already taken, or were currently enrolled in, a health course. For example, topics such as sexual health and drug abuse are some of the eHealth topics most commonly searched by college students and are also frequently covered topics in introductory health courses at colleges and universities [30].

White students reported lower rates of eHealth usage than Asian students or black students. This finding adds to the mixed results from previous studies about differences among individuals from different races/ethnicities and their likelihood of using eHealth [31]. Black students also reported more barriers in comparison to white students when it comes to talking to their medical providers about health information. Although patients tend to have a stronger relationship with providers from their own race or ethnicity, most racial/ethnic minorities in the US do not see providers with the same racial or ethnic identity [32]. Prior studies have highlighted that race may impact the quality of communication in patient-provider relationships [32]. In addition, generational trauma and perceived microaggressions can play a huge role in patient trust and comfort during medical appointments and is commonly found in black populations due to prior medical mistreatment of that community [33,34]. It is possible that minority students used eHealth sources at a higher rate than white students because they were less comfortable with their provider and felt more frequent barriers to sharing health information with their provider [34]. Thus, minority students may try to reduce the need to visit their provider by accessing health information online. This finding highlights a topic deserving further study.

Moreover, students reported feeling more comfortable meeting with their medical provider when their gender identity matched that of their provider. This comfort may stem from empathy as they perceive their medical provider to better understand what they are going through than if their provider is of a different gender identity [35]. Previous literature has shown that patients who share similar characteristics as their medical provider are more likely to trust and be comfortable with that medical provider because of shared experiences, but it has been shown to matter more to female patients than male patients [36,37]. Thus, our study further highlights the importance of having providers available from diverse gender and racial/ethnic identities.

Within the past few decades, medical schools have incorporated cultural competency training to their curriculum because schools want culturally sensitive future physicians who can bring awareness to health disparities [38]. This training is aimed to improve physician-patient communication, collaboration, and patient satisfaction [39–41]. Future research will be needed to see if this improved training reduces the barriers that minority groups experience when seeking health care.

Like previous studies which highlighted that undergraduate students had a perceived eHealth literacy score average around three on the eHEALS instrument [10,25,27,42], these respondents had an average score just under three. This score indicates that the students generally agreed that they could find and use eHealth resources, but they did not feel strongly confident in their ability to do so. Interviews supported the idea that students have doubts about their eHealth literacy. The perceived eHealth literacy scores did not vary based on student major or whether respondents had taken an introductory health course at college or university. Thus, the introductory health courses offered in higher education may not be sufficient in giving students confidence to properly find and evaluate health information online. Consequently, our findings suggest that additional educational resources need to be made available to help undergraduate students confidently access quality health information on the topics about which they most often search online (e.g., sexual health and drug use).

These survey respondents did not perceive that their use of online health resources influenced their patient-provider relationship. On the contrary, interviewees reported that there had been a change in their relationship with their medical provider due to the transition to online appointments in place of in-person appointments due to the COVID-19 pandemic. This timely insight into student perceptions show that undergraduates felt that the lack of a face-to-face meeting with their physician caused communication issues between the patient and provider and reduced their access to the provider as a source of health information. Whether this discomfort with telehealth is a result of pandemic conditions, a transient reaction to the sudden increase in telehealth, or a longer-term hesitation is not yet known.

These students most frequently wanted a “counselor/advisor” model of relationship with their medical provider. This result differs from previous literature which has stated that adults commonly have a “guardian/paternalistic” relationship model with their provider [43]. The shift in the patient-provider relationship may be influenced by the recent increase in accessibility to health information leading to a desire for greater patient autonomy. The “counselor/advisor” relationship model reflects shared decision making where patients help make decisions about their treatment [44]. Ehealth usage may challenge the traditional dynamic between a patient and provider because as patients take more responsibility, they allocate less decision making to the provider [43]. Future studies will be needed to determine if the desire for a “counselor/advisor” relationship is common to users of eHealth or reflects a difference between undergraduate populations and other adult populations.

Most of these undergraduate students indicated that they were comfortable meeting with their providers, especially if they had a primary care provider. Thus, these respondents may be less likely to delay or avoid seeing a health care professional, when needed, or withhold health information from their medical provider. Comfort with their provider may mean their health conditions are less likely to go untreated or undiagnosed. Students may be more likely to avoid providers during the COVID-19 pandemic, however, due to a desire to avoid telehealth appointments. Future research will be needed to determine if students have delayed medical appointments during the pandemic, and if so, whether that has long-term health consequences.

This research study had several limitations. All data were self-reported by students and do not include independent tracking of eHealth usage or student interactions with their providers. In addition, similar to previous studies, students’ competency accessing and using online health information was not tested directly, only students’ perception of their eHealth literacy was. The study population came from a single university, which limits the inferences that can be made. The interview sample sizes were small and thus the results obtained from them may not be representative of the entire population. Finally, these data were collected during the worldwide COVID-19 pandemic and may be influenced by this atypical event. Further research would benefit from surveying undergraduates at other universities, having additional eHealth literacy data that is not only based solely on student perception, and collecting data in non-pandemic times.

## Conclusion

eHealth has made medical information accessible for many populations, including undergraduate students. Undergraduate students reported using eHealth at least occasionally and felt able to find and understand eHealth sources, though they were not strongly confident in their ability to do so and were skeptical of telehealth appointments. Thus, students may benefit from having additional training to boost their eHealth literacy. Students in this study most frequently reported wanting a patient-provider relationship similar to the “counselor/advisor” model, in which patients make health decisions in partnership with their provider. Despite access to eHealth, student race, ethnicity, and gender identity impacted students’ comfort with their provider, demonstrating that health disparities still exist with this population. Students prefer gaining information from providers, particularly those who share their same gender identity or race, over relying on eHealth sources. Thus, having diverse, culturally competent medical providers may help students to develop a positive relationship with their provider.

## Supporting information

S1 Table Interview codebook. (PDF)

S2 Table Number and percent of survey respondents indicating each ranking for sources they prioritize when making health decisions. (PDF)

S3 Table Means of each eHEALS question and the mean eHEALS score of students. (PDF)

S1 File Survey questions. (PDF)

S2 File Interview questions. (PDF)

S3 File Survey data. (CSV)

## References

[1] Eysenbach G. What is e-health? J Med Internet Res. 2001 Jun 18;3(2):e833. 10.2196/jmir.3.2.e20 PMC1761894 11720962

[2] Hirvonen N, Enwald HP, Känsäkoski H, Eriksson-Backa K, Nguyen H, Huhta AM, et al. Older adults’ views on eHealth services: a systematic review of scientific journal articles. Int. J. Med Inform. 2020 Mar 1;135:104031. doi: 10.1016/j.ijmedinf.2019.104031 31918340

[3] Andreassen HK, Trondsen M, Kummervold PE, Gammon D, Hjortdahl P. Patients who use e-mediated communication with their doctor: new constructions of trust in the patient-doctor relationship. Qual Health Res. 2006 Feb;16(2):238–48. doi: 10.1177/1049732305284667 16394212

[4] Asan O, Crotty B, Nagavally S, Egede LE. Patient centered communication and e-health information exchange patterns: findings from a national cross-sectional survey. IEEE J Transl Eng Health Med. 2018 Dec 5;7:1–7. doi: 10.1109/JTEHM.2018.2884925 30588412 PMC6302924

[5] Bidmon S, Terlutter R. Gender differences in searching for health information on the internet and the virtual patient-physician relationship in Germany: exploratory results on how men and women differ and why. J Med Internet Res. 2015 Jun 22;17(6):e4127. doi: 10.2196/jmir.4127 26099325 PMC4526954

[6] Britt RK, Collins WB, Wilson K, Linnemeier G, Englebert AM. eHealth literacy and health behaviors affecting modern college students: a pilot study of issues identified by the American College Health Association. J Med Internet Res. 2017;19(12):e392. doi: 10.2196/jmir.3100 29258979 PMC5750421

[7] De Rosis S, Barsanti S. Patient satisfaction, e-health and the evolution of the patient–general practitioner relationship: evidence from an Italian survey. Health Policy. 2016 Nov;120(11):1279–92. doi: 10.1016/j.healthpol.2016.09.012 27836231

[8] Mitsutake S, Shibata A, Ishii K, Oka K. Associations of eHealth literacy with health behavior among adult internet users. J Med Internet Res. 2016;18(7):e192. doi: 10.2196/jmir.5413 27432783 PMC4969548

[9] Norman CD, Skinner HA. eHEALS: The eHealth literacy scale. J Med Internet Res. 2006 Nov 14;8(4):e27. doi: 10.2196/jmir.8.4.e27 17213046 PMC1794004

[10] Britt RK, Hatten KN. Need for cognition and electronic health literacy and subsequent information seeking behaviors among university undergraduate students. SAGE Open. 2013 Jan 1;3(4):2158244013508957.

[11] Hanik B, Stellefson M. E-Health literacy competencies among undergraduate health education students: a preliminary study. Int Electron J Health Educ. 2011;14(1).

[12] Kim S-H, Son Y-J. Relationships between eHealth literacy and health behaviors in Korean adults. Comput Inform Nurs. 2017 Feb;35(2):84. doi: 10.1097/CIN.0000000000000255 27258808

[13] Vraga EK, Bode L. Using expert sources to correct health misinformation in social media. Sci Commun. 2017 Oct;39(5):621–45.

[14] Cline RJ, Haynes KM. Consumer health information seeking on the Internet: the state of the art. Health Educ Res. 2001 Dec 1;16(6):671–92. doi: 10.1093/her/16.6.671 11780707

[15] Brennen JS, Simon F, Howard PN, Nielsen RK. Types, sources, and claims of COVID-19 misinformation. Reuters Institute. 2020 Apr 7;7(3):1.

[16] Contreras CM, Metzger GA, Beane JD, Dedhia PH, Ejaz A, Pawlik TM. Telemedicine: patient-provider clinical engagement during the COVID-19 pandemic and beyond. J Gastrointest Surg. 2020 Jul;24(7):1692–7. doi: 10.1007/s11605-020-04623-5 32385614 PMC7206900

[17] Thomas EE, Haydon HM, Mehrotra A, Caffery LJ, Snoswell CL, Banbury A, et al. Building on the momentum: sustaining telehealth beyond COVID-19. J Telemed Telecare. 2020 Sep 26:1357633X20960638. doi: 10.1177/1357633X20960638 32985380

[18] Emanuel EJ, Emanuel LL. Four models of the physician-patient relationship. JAMA. 1992 Apr 22;267(16):2221–6. 1556799

[19] Tan SS, Goonawardene N. Internet health information seeking and the patient-physician relationship: a systematic review. J Med Internet Res. 2017;19(1):e9. doi: 10.2196/jmir.5729 28104579 PMC5290294

[20] Haywood C, Lanzkron S, Ratanawongsa N, Bediako SM, Lattimer L, Powe NR, et al. The association of provider communication with trust among adults with Sickle Cell Disease. J Gen Intern Med. 2010 Jun;25(6):543–8. doi: 10.1007/s11606-009-1247-7 20195785 PMC2869431

[21] Nural N, Alkan S. Identifying the factors affecting comfort and the comfort levels of patients hospitalized in the coronary care unit. Holist Nurs Pract. 2018 Jan;32(1):35–42. doi: 10.1097/HNP.0000000000000245 29210876

[22] Lin RJ. Commentary on “Primary Care Providers’ Comfort Levels in Caring for Patients With Sickle Cell Disease”. South Med J. 2015 Sep;108(9):537–8. doi: 10.14423/SMJ.0000000000000332 26332478 PMC7062503

[23] Whiteman LN, Haywood C, Lanzkron S, Strouse JJ, Feldman L, Stewart RW. Primary care providers’ comfort levels in caring for patients with sickle cell disease. South Med J. 2015 Sep;108(9):531–6. doi: 10.14423/SMJ.0000000000000331 26332477

[24] Santana S, Lausen B, Bujnowska-Fedak M, Chronaki CE, Prokosch H-U, Wynn R. Informed citizen and empowered citizen in health: results from an European survey. BMC Fam Pract. 2011 Apr 16;12(1):20. doi: 10.1186/1471-2296-12-20 21496309 PMC3101118

[25] Robb M, Shellenbarger T. Influential factors and perceptions of eHealth literacy among undergraduate college students. Online J Nurs Inform. 2014 Oct 1;18.

[26] Brown CA, Dickson R. Healthcare students’ e-literacy skills. J Allied Health. 2010 Jan 1;39(3):179–84. 21174023

[27] Tsukahara S, Yamaguchi S, Igarashi F, Uruma R, Ikuina N, Iwakura K, et al. Association of ehealth literacy with lifestyle behaviors in university students: questionnaire-based cross-sectional study. J Med Internet Res. 2020 Jun 24;22(6):e18155. doi: 10.2196/18155 32579126 PMC7381004

[28] Chung S-Y, Nahm E-S. Testing reliability and validity of the eHealth Literacy Scale (eHEALS) for older adults recruited online. Comput Inform Nurs. 2015 Apr;33(4):150–6. doi: 10.1097/CIN.0000000000000146 25783223 PMC4442634

[29] van der Vaart R, van Deursen AJ, Drossaert CH, Taal E, van Dijk JA, van de Laar MA. Does the eHealth Literacy Scale (eHEALS) measure what it intends to measure? Validation of a Dutch version of the eHEALS in two adult populations. J Med Internet Res. 2011 Nov 9;13(4):e86. doi: 10.2196/jmir.1840 22071338 PMC3222202

[30] Escoffery C, Miner KR, Adame DD, Butler S, McCormick L, Mendell E. Internet use for health information among college students. J Am Coll Health. 2005 Jan;53(4):183–8. doi: 10.3200/JACH.53.4.183-188 15663067

[31] Reiners F, Sturm J, Bouw LJW, Wouters EJM. Sociodemographic factors influencing the use of eHealth in people with chronic diseases. Int J Environ Res Public Health. 2019 Feb;16(4). doi: 10.3390/ijerph16040645 30795623 PMC6406337

[32] Beach MC, Saha S, Korthuis PT, Sharp V, Cohn J, Wilson IB, et al. Patient–provider communication differs for black compared to white HIV-Infected patients. AIDS Behav. 2011 May;15(4):805–11. doi: 10.1007/s10461-009-9664-5 20066486 PMC2944011

[33] Goosby BJ, Heidbrink C. Transgenerational consequences of racial discrimination for African American health. Sociol Compass. 2013 Aug 1;7(8):630–43. doi: 10.1111/soc4.12054 24855488 PMC4026365

[34] Kanter JW, Rosen DC, Manbeck KE, Branstetter HML, Kuczynski AM, Corey MD, et al. Addressing microaggressions in racially charged patient-provider interactions: a pilot randomized trial. BMC Med Educ. 2020 Mar 24;20(1):88. doi: 10.1186/s12909-020-02004-9 32209082 PMC7092438

[35] Nolen HA, Moore JX, Rodgers JB, Wang HE, Walter LA. Patient preference for physician gender in the emergency department. Yale J Biol Med. 2016 Jun 27;89(2):131–42. 27354840 PMC4918861

[36] Derose KP, Hays RD, McCaffrey DF, Baker DW. Does physician gender affect satisfaction of men and women visiting the emergency department? J Gen Intern Med. 2001 Apr;16(4):218–26. doi: 10.1046/j.1525-1497.2001.016004218.x 11318922 PMC1495193

[37] Mainous AG, Goodwin MA, Stange KC. Patient-physician shared experiences and value patients place on continuity of care. Ann Fam Med. 2004 Sep;2(5):452–4. doi: 10.1370/afm.84 15506580 PMC1466737

[38] Swanberg SM, Abuelroos D, Dabaja E, Jurva S, Martin K, McCarron J, et al. Partnership for diversity: a multidisciplinary approach to nurturing cultural competence at an emerging medical school. Med Ref Serv Q. 2015 Oct 2;34(4):451–60. doi: 10.1080/02763869.2015.1082379 26496399

[39] Deliz JR, Fears FF, Jones KE, Tobat J, Char D, Ross WR. Cultural competency interventions during medical school: a scoping review and narrative synthesis. J Gen Intern Med. 2020 Feb;35(2):568–77. doi: 10.1007/s11606-019-05417-5 31705475 PMC7018865

[40] Kripalani S, Bussey-Jones J, Katz MG, Genao I. A prescription for cultural competence in medical education. J Gen Intern Med. 2006 Oct;21(10):1116–20. doi: 10.1111/j.1525-1497.2006.00557.x 16836623 PMC1831630

[41] LeBlanc TW, Hesson A, Williams A, Feudtner C, Holmes-Rovner M, Williamson LD, et al. Patient understanding of medical jargon: a survey study of U.S. medical students. Patient Educ Couns. 2014 May 1;95(2):238–42. doi: 10.1016/j.pec.2014.01.014 24525222

[42] Tubaishat A, Habiballah L. eHealth literacy among undergraduate nursing students. Nurse Educ Today. 2016 Jul 1;42:47–52. doi: 10.1016/j.nedt.2016.04.003 27237352

[43] Grünloh C, Myreteg G, Cajander Å, Rexhepi H. “Why do they need to check me?” Patient participation through eHealth and the doctor-patient relationship: qualitative study. J Med Internet Res. 2018 Jan 15;20(1). doi: 10.2196/jmir.8444 29335237 PMC5789160

[44] Bernabeo E, Holmboe ES. Patients, providers, and systems need to acquire a specific set of competencies to achieve truly patient-centered care. Health Aff (Millwood). 2013 Feb;32(2):250–8. doi: 10.1377/hlthaff.2012.1120 23381517

