# Supplementary material for: Understanding undergraduate students’ eHealth usage and views of the patient-provider relationship
Source: PLoS One. 2022 Apr 14;17(4):e0266802. doi: 10.1371/journal.pone.0266802 (PMC9009692; doi:10.1371/journal.pone.0266802)
Supplement: S1 Table — (PDF) [file pone.0266802.s001.pdf]

S1 Table: Interview Codebook

Code	Description	Examples
PANDEMIC	Any relation of eHealth to telehealth or the current pandemic	• Seeing less of the physician<br>• Easier to have a relationship in person than over the phone<br>• Harder to make decisions without physician there
SOURCE: INT	Mention of medical information sources from the internet	• Electronics<br>• Medical journals/publications<br>• Internet
SOURCE: PHY	Mentioning of medical information sources from a physician	• Doctor tells me what I need to hear<br>• Getting health information directly from doctor first (prioritized)<br>• Mentioning physicians offering guidance or advice can be considered as a source of information
RS: PHY	Relationship with physician being a factor (whether it's short or long)	• Knowing them (the physician) for a long time is better than someone new<br>• Seeing them for a long time makes talking about stuff easier<br>• Mentioning being more comfortable with their doctor since they have known them for a long time or know more about them
PRO	Mention of physicians being 'professional' or being more knowledgeable	• They are the experts- they should know what I need to do<br>• I use the internet for information, but they (the physician) know more accurate stuff<br>• Seeing the physician as knowledgeable because

		of their occupation and/or education
+ ENCOURAGE	Physician offering positive encouragement for eHealth information	• They always tell me to look things up on my own<br>• Physician encourages conversation about information gathered<br>• eHealth information is shared without contest from physician
- ENCOURAGE	Physician offering negative encouragement for eHealth information	• They (the physician) don't tell me to look up information at home<br>• Physician not mentioning or bringing up eHealth at all<br>• Physician claiming they know more than online sources
COMFORT	Comfortability being a factor in information sharing or physician relationship	• I prefer someone I know instead of a new doctor<br>• Sharing demographics with a physician – gender, race, age, etc.
INFO DOUBT	Mention of misinformation/inaccurate information from medical sources	• Not everything you look up is right<br>• Not sure if the information I find is accurate<br>• Mentioning need clarification or reassurance with information they gather

2
