# Supplementary material for: Understanding undergraduate students’ eHealth usage and views of the patient-provider relationship
Source: PLoS One. 2022 Apr 14;17(4):e0266802. doi: 10.1371/journal.pone.0266802 (PMC9009692; doi:10.1371/journal.pone.0266802)
Supplement: S2 Table — (PDF) [file pone.0266802.s002.pdf]

S2 Table: Number and percent of survey respondents indicating each ranking for sources they prioritize when making health decisions (1 = top priority, 8 = lowest priority).

**Ranking**	**Family**		**Ranking**	**Social Media**	
	Number	%		Frequency	%
1	118	22.4	1	2	0.4
2	207	39.3	2	5	0.9
3	140	26.6	3	26	4.9
4	48	9.1	4	32	6.1
5	9	1.7	5	113	21.4
6	2	0.4	6	252	47.8
7	0	0	7	89	16.9
8	3	0.6	8	8	1.5
	**Friends**			**Health Information Websites**	
	Frequency	%		Frequency	%
1	6	1.1	1	15	2.8
2	35	6.6	2	158	30
3	111	21.1	3	97	18.4
4	194	36.8	4	58	11
5	132	25	5	112	21.3
6	36	6.8	6	79	15
7	12	2.3	7	8	1.5
8	1	0.2	8	0	0
	**Partner**			**Television**	
	Frequency	%		Frequency	%
1	3	0.6	1	0	0
2	40	7.6	2	1	0.2
3	125	23.7	3	4	0.8
4	141	26.8	4	11	2.1
5	133	25.2	5	19	3.6
6	45	8.5	6	100	19
7	29	5.5	7	365	69.3
8	11	2.1	8	27	5.1
	**Medical Source**			**Other**	
	Frequency	%		Frequency	%
1	390	72.1	1	3	0.6

2	72	13.7	2	9	1.7
3	22	4.2	3	2	0.4
4	40	7.6	4	3	0.6
5	6	1.1	5	3	0.6
6	4	0.8	6	9	1.7
7	3	0.6	7	21	4
8	0	0	8	477	90.5
