# Supplementary material for: Understanding undergraduate students’ eHealth usage and views of the patient-provider relationship
Source: PLoS One. 2022 Apr 14;17(4):e0266802. doi: 10.1371/journal.pone.0266802 (PMC9009692; doi:10.1371/journal.pone.0266802)
Supplement: S3 Table — (PDF) [file pone.0266802.s003.pdf]

S3 Table. Means of each eHEALS question and the mean eHEALS score of students. See Table 1 in their original publication for the items: Norman CD, Skinner HA. eHEALS: The eHealth Literacy Scale. J Med Internet Res. 2006 Nov 14;8(4):e27.

	**Mean**
Q1.	2.83
Q2.	2.79
Q3.	2.76
Q4.	2.75
Q5.	2.72
Q6.	2.69
Q7.	2.94
Q8.	2.22
**Mean eHEALS score:**	2.71
