# Supplementary material for: Understanding undergraduate students’ eHealth usage and views of the patient-provider relationship
Source: PLoS One. 2022 Apr 14;17(4):e0266802. doi: 10.1371/journal.pone.0266802 (PMC9009692; doi:10.1371/journal.pone.0266802)
Supplement: S1 File — (PDF) [file pone.0266802.s004.pdf]

S1 File: Survey Questions

Note: Likert scale survey questions were reversed before analyses to ease interpretation. For example, higher eHealth usage on the survey is indicated by lower Likert scales but in the analysis stage higher eHealth usage is reflected as a higher score.

1) What is your age?
   a) 18-20
   b) 21-24
   c) 25+
2) What is your class rank? (Based on semester hours)
   a) Freshman
   b) Sophomore
   c) Junior
   d) Senior
3) What is your gender identity?
   a) Female
   b) Male
   c) Other
   d) Do not wish to answer
4) What is your racial or ethnic identification?
   a) American Indian or Alaska Native
   b) Asian
   c) Black or African American

d) Hispanic or Latino

e) Native Hawaiian or Other Pacific Islander

f) White

g) Other

h) Do not wish to answer

5) What is your major?

a) Enter data

6) Do you intend to pursue a health related career (medical, dental, physician assistant, physical therapy, occupational therapy, etc.)?

a) Yes

b) No

7) Have you taken an introductory health course at the university/college level (i.e., Health 1000 at ECU)?

a) Yes

b) No

c) Currently enrolled

8) Do you have any pre-existing health condition?

a) Yes

b) No

9) Where do you get your health information?

a) Online source

i) Enter if available

b) Personal source (family, friend, etc.)

c) Medical source (family doctor, physician, etc.)

d) School source (health center, health class, teacher, etc.)

10) When you look up health information, who is it for usually? (Mark all that apply)

a) Self

b) Partner

c) Friend

d) Family

e) Other

11) How often do you use online sources when making decisions about your health?

a) All the time

b) Almost every time

c) Occasionally

d) Very seldom

e) Not at all

12) Rank what sources you prioritize when making health decisions (1 = top priority, 8 = lowest priority)

a) Family

b) Friends

c) Partner

d) Medical provider

e) Online source: Social media

f) Online source: Health information websites (i.e., WebMD)

g) Television

h) Other

i) Enter data

13) How accurate do you believe your source of health information is?

a) Very accurate

b) Somewhat accurate

c) Average

d) Not every accurate

e) Not accurate at all

14) Do you believe you have the skills to understand and utilize the health information you gather from the internet?

a) Yes

b) No

15) Do you feel comfortable when you are meeting with a physician?

a) All the time

b) Almost every time

c) Occasionally

d) Very seldom

e) Not at all

16) What model of relationship do you believe most patient-physicians relationships follow?

a) Guardian/Paternalistic: Patient submits to objective/non-personal values while the provider acts as a guardian and decides all actions for the patient

b) Counselor/Advisor: Patient has some personal understanding relevant to medical care while the provider acts as a counselor or adviser

c) Technical Expert: Patient has choice of, and control over medical care while the provider acts as competent technical expert

d) Friend/Teacher: Patients have moral self-development relevant to medical care while the provider acts as a friend or teacher

17) What model of relationship would you like to have with your physician?

a) Guardian/Paternalistic: Patient submits to objective/non-personal values while the provider acts as a guardian and decides all actions for the patient

b) Counselor/Advisor: Patient has some personal understanding relevant to medical care while the provider acts as a counselor or adviser

c) Technical Expert: Patient has choice of, and control over medical care while the provider acts as competent technical expert

d) Friend/Teacher: Patients have moral self-development relevant to medical care while the provider acts as a friend or teacher

18) Has your ability to access online health sources changed your relationship with your physician or medical provider?

a) Yes

b) Somewhat

c) No

19) When/if you see a medical provider, do you consult with them about the information you find through online sources?

a) Yes

b) No

20) When/if you see a medical provider, do you feel confident/comfortable when telling them about the information you find through online health sources?

a) Yes

b) No

21) Does the gender of your physician influence your level of trust during appointments?

a) Yes

b) Somewhat

c) No

22) What is the gender identity of your physician?

a) Male

b) Female

c) Other

d) Do not wish to answer

23) Are there barriers that restrict or prohibit you from discussing health information you find with your physician?

a) All the time

b) Almost every time

c) Occasionally

d) Very seldom

e) Not at all

   i) Please explain

24) Are you encouraged by your physician to research health information on your own?

a) All the time

b) Almost every time

c) Occasionally

d) Very seldom

e) Not at all

Items from Norman and Skinner (2006) were included here. See Table 1 in their original publication for the items: Norman CD, Skinner HA. eHEALS: The eHealth Literacy Scale. J Med Internet Res. 2006 Nov 14;8(4):e27.
