# Supplementary material for: Understanding undergraduate students’ eHealth usage and views of the patient-provider relationship
Source: PLoS One. 2022 Apr 14;17(4):e0266802. doi: 10.1371/journal.pone.0266802 (PMC9009692; doi:10.1371/journal.pone.0266802)
Supplement: S2 File — (PDF) [file pone.0266802.s005.pdf]

S2 File: Interview Questions

Verbal consent requested. Welcome and brief explanation of the format of the interview: Open discussion that will last approximately 20-30 minutes with 6 starting questions and follow up questions, as needed.

Question 1: How do you define eHealth?

Question 2: If you use eHealth, do you feel in control of your health/empowered?

Question 3: Do you feel encouraged to search eHealth information from your physician?

Question 4: What factors contribute to whether you discuss eHealth information with your physician?

Question 5: What model of patient-physician relationship do you believe you have with your physician and why?

Question 6: Would you change that relationship model and why?
